# Maternal obesity and the embryonic rewiring of feeding circuits: Beyond the hypothalamus

**DOI:** 10.1016/j.molmet.2026.102385

**Published:** 2026-05-23

**Authors:** Dionysios V. Chartoumpekis, Aristea Psilopanagioti

**Affiliations:** Division of Endocrinology, Department of Internal Medicine, School of Medicine, University of Patras, Patras, GR-26504, Greece

**Keywords:** Maternal obesity, Fetal reprogramming, Feeding circuits, Brainstem, Mesocorticolimbic reward system, Hypothalamus

## Abstract

Maternal obesity disrupts fetal hypothalamic development by inducing structural changes in feeding circuits, such as reduced hypothalamic precursor proliferation, blunted energy-status sensing, and aberrant wiring of melanocortin pathways, ultimately promoting long-term obesity risk in offspring. Although this hypothalamic reprogramming has dominated research, emerging evidence supports a broader, distributed model involving both brainstem interoceptive circuits and mesocorticolimbic reward-control systems. Maternal obesity is associated with impaired white matter development in offspring, as well as compromised functional connectivity, abnormal orexigenic and anorexigenic signaling, hypothalamic inflammation, and epigenetic alterations in neurodevelopmental genes. Human neuroimaging studies demonstrate altered network connectivity in neonates associated with maternal adiposity. However, the mechanism through which dysregulated maternal signals ultimately reach and reprogram the developing brain remains largely elusive. The present review aims to elucidate the mechanisms by which maternal overnutrition drives the embryonic rewiring of feeding circuits beyond the hypothalamus, highlighting the susceptibility of these extra-hypothalamic neural networks to obesogenic programming. Future research should prioritize investigating the effects of maternal obesity on fetal cytoarchitecture and function in feeding-related neuroanatomical circuits, including brainstem interoceptive nuclei and mesocorticolimbic reward–control pathways. Elucidating these developmental neuro-metabolic changes may offer the opportunity to establish early intervention measures for preserving offspring metabolic health.

## Highlights

• Maternal obesity reprograms extra-hypothalamic feeding circuits in offspring.
• Brainstem and mesocorticolimbic circuits show sex-specific ontogenetic shifts.
• Human neonates exhibit altered white matter integrity linked to maternal BMI.
• Neuroinflammation and epigenetic modifications drive distributed neural defects.
• Early extra-hypothalamic rewiring elevates long-term offspring obesity risk.

## 1 Introduction

Maternal obesity reflects a systemic physiological state characterized by altered circulating factors and metabolic signaling, low-grade systemic inflammation, and placental adaptations, such as lipotoxicity, inflammation, and disruption of vascular development, that collectively influence the intrauterine environment [1]. Epidemiological studies suggest that intrauterine exposure to maternal overnutrition or obesity increases susceptibility of offspring to obesity and has long-term metabolic complications that may persist into adulthood [2,3]. These pathophysiological alterations are clinically important, as obesity is more difficult to treat in children than in adults and increases the long-term risk for several severe diseases [4,5]. In rodent models, it is well established that exposure to high-fat and high-sugar diet prior to gestation, during gestation or during lactation results in the phenotypic characteristics of metabolic syndrome in offspring [[6], [7], [8], [9], [10]].

It has been suggested that this predisposition is based on altered neurodevelopmental programming of the hypothalamus, such as reduced hypothalamic precursor proliferation, altered energy-status sensing and disrupted wiring of melanocortin pathways, during critical periods of fetal development, the first trimester being the most critical period for programming susceptibility to obesity, in humans [2,11]. The human fetal brain begins to develop during the third gestational week and grows rapidly, especially in the third trimester [12]. In the human hypothalamus, neurogenesis and neuronal differentiation as well as basic connectivity patterns and formation of functional connectivity starts in the first trimester and extends into the third trimester of pregnancy [[13], [14], [15], [16], [17]]. It is important to note that although hypothalamic connectivity in humans is established prenatally, murine hypothalamic development follows a different temporal trajectory. Specifically, murine hypothalamic neurons appear between embryonic days 12 and 18 and functional connectivity between hypothalamic nuclei is established during the first four weeks of the post-natal period [13,[18], [19], [20]].

Offspring hypothalamic programming due to *in utero* exposure to maternal obesity involves hypothalamic inflammation, mitochondrial dysfunction, oxidative stress, and changes in neuronal proliferation and differentiation, as well as in the expression pattern of clock genes. For a comprehensive review of mechanisms mediating the impact of maternal obesity on offspring early hypothalamic programming see Furigo et al. [11] and Zhang et al. [21].

Thus, converging data indicate that maternal diet fundamentally reprograms hypothalamic structure and function during the critical developmental period for the establishment of feeding circuitry [11,21]. However, the neural control of energy homeostasis extends beyond these circuits, involving a distributed network of neuronal and non-neuronal substrates [22]. It is now well established that the hypothalamus operates within a broader neuroanatomical axis, integrating signals from cortical and subcortical regions —including the orbitofrontal and insular cortices, the hippocampus, the extended amygdala, the mesocorticolimbic system, and the brainstem— to orchestrate complex, motivated feeding behaviors [23,24].

The dorsal vagal complex (DVC) —a key brainstem structure for energy balance regulation comprising the area postrema, nucleus tractus solitarius (NTS) and dorsal motor nucleus of the vagus (DMNX)— integrates vagus-mediated mechanical and chemical gastrointestinal signals, circulating energy-related humoral signals, and descending projections to and from midbrain and forebrain nuclei to regulate food intake [3,24]. In addition to neuronal circuits, non-neuronal brainstem cell populations are increasingly recognized for their role in the regulation of feeding behavior [22,25]. According to MacDonald et al. [25], high-fat feeding in adult mice upregulates glial fibrillary acidic protein expression and induces morphological changes in NTS astrocytes. Furthermore, chemogenetic activation of astrocytes in the DVC reversibly reduced food intake, acting as a compensatory response to nutritional excess [25].

Concurrently, feeding behavior extends beyond hypothalamic and brainstem homeostatic circuits, to encompass hedonic mechanisms that profoundly shape the executive, emotional, and mnemonic processing of food intake [23]. The mesocorticolimbic circuitry, comprising the ventral tegmental area (VTA), nucleus accumbens, ventral pallidum, dorsal striatum, prefrontal and orbitofrontal cortices, hippocampus, and amygdala, constitutes a critical extra-hypothalamic network regulating reward-based food intake [23,[26], [27], [28]]. These circuits encode the “liking” (hedonic impact) of palatable foods through opioid and endocannabinoid signaling, the motivational “wanting” (incentive salience) via dopaminergic pathways, and the associative learning processes that reinforce feeding behavior [23,[26], [27], [28], [29], [30], [31]]. Because these crucial brainstem and mesocorticolimbic networks undergo extensive development and functional maturation during fetal and early postnatal life, they represent highly vulnerable targets for maternal obesogenic programming [[32], [33], [34], [35], [36], [37], [38], [39]].

Although the majority of evidence has focused on the crucial role of hypothalamic reprogramming in the metabolism of the offspring exposed to maternal obesity, additional research is still needed to clarify the vulnerability of these broader, neuroanatomically distributed brain structures. The present review examines the impact of maternal overnutrition and obesity on offspring brain development, focusing on neural circuits beyond the well-studied hypothalamus. By synthesizing the currently limited experimental data on the brainstem and the mesocorticolimbic reward circuitry, we aim to highlight the mechanisms driving the embryonic rewiring of these extra-hypothalamic networks.

## 2 The impact of maternal obesity on brainstem

Consisting of heterogeneous populations of neurons, brainstem circuits integrate homeostasis-related humoral and neural signals and modulate feeding behavior [40,41]. However, there are very few data on the impact of maternal obesity on the cytoarchitecture of the developing brainstem. Interestingly, the brainstem matures relatively early in fetal development. Vagal neurocircuitry starts to develop from embryonic day 13 and the DMNX completes its maturation by embryonic day 18 [32,33,35]. In rodents, although neuropeptide Y (NPY) neurons from the arcuate nucleus innervate the paraventricular hypothalamic nucleus at post-natal day 10–11, brainstem NPY-positive neuronal fibers projecting to paraventricular nucleus are present from the post-natal day 2 [42]. In humans, at 6 weeks, the rhombencephalon will further divide into the metencephalon, which will form the pons and cerebellum, and the myelencephalon that will produce the medulla [43,44]. Thus, brainstem circuits related to food intake, including sucking, swallowing, and taste, develop before hypothalamic, and are possibly more vulnerable to environmental insults such as maternal overnutrition.

Maternal exposure to high-fat and high-sucrose diets significantly impacts the plasticity of non-neuronal cells within the rat offspring DVC [3]. Even brief *in utero* and early postnatal exposure to such diets alters offspring body weight, plasma insulin and leptin levels, while inducing glial changes characterized by astrocyte reactivity, microglia activation, and a reduction in vimentin density of tanycytes [3]. The acute responsiveness of this region to dietary lipids is further evidenced by adult models, where a mere 12-hour exposure to a high-fat diet triggers hyperphagia correlated with increased astrocyte number and morphological complexity in the mouse NTS [25]. Collectively, these observations suggest that DVC glia serve as rapid metabolic sensors and that developmental overnutrition may recalibrate their structural and functional response to dietary fat, potentially driving long-term obesity risk.

Exposure to a maternal high-fat diet impairs central vagal neurotransmission and induces changes in autonomic neurocircuits. Specifically, in rat models, a perinatal high-fat diet increases gamma-aminobutyric acid (GABA)-mediated inhibitory tone, impairs glutamate-mediated excitatory inputs, and decreases the spontaneous firing rate of neurons in the DMNX [[45], [46], [47]]. Such alterations in central vagal neurocircuits decrease the excitability of gastric-projecting neurons in the DMNX and lead to dysregulation of neurotransmitter release and diminished gastric tone and motility prior to the development of obesity in the offspring [48].

In addition to alterations in the DMNX, a maternal high-fat diet down-regulated the expression of metabotropic glutamate_2/3_ receptor (mGlu_2/3_R) —but not *N*-methyl-d-aspartate receptor subunit 1— in the caudal ventrolateral medulla and the NTS of male rat offspring, despite inducing no change in body weight until early adulthood [49]. Furthermore, a reduction in GABA_A_ receptor (GABA_A_R)-expressing tyrosine hydroxylase-immunoreactive cells was observed in the rostral ventrolateral medulla of male rats, findings that implicate autonomic imbalance and increased sympathetic tone. Notably, these alterations in receptor expression exhibited sex-dependent effects, as no such changes were observed in female offspring [49].

Furthermore, maternal high-fat diet during pregnancy disrupts the fetal serotonergic system in nonhuman primates by increasing the expression of tryptophan hydroxylase 2, the rate-limiting enzyme for serotonin synthesis, as well as of serotonin 1A receptor subtype (5-HT_1A_R), an inhibitory autoreceptor, in the rostral raphe nuclei, which are the primary source of hypothalamic serotonin [50,51]. Given that serotonergic signaling is critically implicated in both homeostatic and hedonic brain circuitries, its developmental disturbance may play a role in the susceptibility of the offspring to metabolic programming [52].

## 3 The impact of maternal obesity on reward control systems

In humans, it has been suggested that the neuronal circuitry regulating reward-based aspects of feeding behavior develops in early childhood, gradually overriding the homeostatic mechanisms of food intake (such as the orosensory and visceral systems) [2]. However, the structural foundation for these hedonic networks is established much earlier, during fetal life. Specifically, in the human fetus, dopamine D1 and D2 receptor (D1R and D2R)-positive neurons are clearly observed as early as 19 gestational weeks [53]. In humans, by 32 weeks of gestation, the dopamine reuptake transporter (DAT) is detectable in the neuropil, neurons, and non-neuronal cells of the basal ganglia, and projection formation, refinement, and maturation of dopaminergic neurons occur during the last trimester of pregnancy [53]. In rodents, although reward-based learning and motivation do not fully emerge until the transition to independent feeding, mesolimbic reward circuits begin forming during gestation [2,[36], [37], [38], [39]]. As these circuits are developing during pregnancy and early post-natal life, they are highly susceptible to maternal nutritional insults. In rodents, maternal intake of an obesogenic diet throughout gestation has been shown to fundamentally reprogram these pathways, correlating with an increased preference for fat in the offspring [6,[54], [55], [56]].

Within the dopaminergic system, a maternal high-fat diet significantly upregulates DAT expression in the nucleus accumbens, hypothalamus, and prefrontal cortex in mice offspring [55,56]. Importantly, DAT expression appears sensitive to the specific timing of the metabolic insult and exhibits profound sex differences. For example, utilizing a mouse embryo transfer model to isolate the gestational environment, Grissom et al. [57] demonstrated that exposure to an obesogenic gestational milieu causes a significant increase in DAT within the VTA of adult males, but a marked decrease in adult females. Furthermore, maternal high-fat diet during lactation reprograms offspring dopaminergic circuitry in VTA and substantia nigra, at multiple functional levels. It alters intrinsic firing properties in midbrain dopaminergic neurons, reduces functional connectivity to downstream targets, increases the excitability of postsynaptic D1 medium spiny neurons (MSNs), while decreasing D2 MSN projections. These neural changes correlate with sexually dimorphic behavioral patterns, including hyperlocomotion in male and increased intake of palatable food in female mice [16]. Consistently, offspring exposed to high-fat diet during the last week of gestation and lactation display altered presynaptic regulation of dopamine in the nucleus accumbens, accompanied by a decrease in dopamine release-regulating D2 receptor expression in VTA, driving an increased motivation for fat-rich rewards [56].

Parallel to these dopaminergic alterations, the opioidergic system is also highly susceptible to maternal obesogenic programming. Particularly, in the rodent offspring nucleus accumbens, a critical hub involved in “liking” and “wanting” of hedonic feeding, it is well established that mu-opioid receptor (MOR) expression is increased, due to hypomethylation [54,[57], [58], [59]]. Using the same embryo transfer model mentioned above to decouple the effects of obesity at conception from the gestational environment, Grissom et al. [57] demonstrated that maternal metabolic status at fertilization alone is sufficient to reprogram offspring reward circuitry. In male mice, pre-gestational and gestational obesity upregulated MOR and preproenkephalin (PENK) across the mesocorticolimbic system (VTA, nucleus accumbens, prefrontal cortex, and hypothalamus) [57]. Conversely, adult female mice exhibited an opposing phenotype, characterized by a downregulation of MOR and PENK specifically within the nucleus accumbens [57]. As MOR and PENK are central to coding the hedonic aspects of food intake [60], these neurochemical alterations drive offspring toward an increased preference for palatable foods. Importantly, Grissom et al. highlighted critical time-dependent differences in these signatures; neurochemical profiles at embryonic day 17.5 (E17.5) often deviated from adult outcomes, such as a transient decrease in forebrain MOR and PENK in male embryos, underscoring the dynamic ontogenetic evolution of maternal programming [57]. Additionally, in male, but not in female, rats, while MOR expression correlates positively with fat intake during the immediate post-weaning period (6 weeks), this relationship is reversed by 3 months of age [54,61]. Highlighting the translational relevance of these networks, Haghighi et al. demonstrated that the minor allele of the mu-opioid receptor gene (*OPRM1*) is associated with higher amygdala volume and lower fat intake in human subjects [62].

In rodent offspring, perinatal exposure to high-fat and/or high-sugar diets modulates the expression of both dopamine and opioid-related genes in reward-associated brain areas and increases animals' preference for highly palatable foods, potentially through impaired reward processing and motivation [[54], [55], [56],^63^]. However, transcriptional analyses of dopamine and opioid circuits remain challenging because of the extensive neuroplastic remodelling that occurs during the peri-weaning period.

Additionally, in mouse models, a maternal high-fat diet prior to and during gestation induces region-specific disruptions in proliferation of neural precursors (showing an increase in the hippocampus and cortex, but a decrease within the dentate gyrus) and reduced apoptosis [64]. In parallel, the differentiation of calretinin-positive neurons within the dentate gyrus of male fetuses was decreased [64]. Calretinin, a calcium-binding protein, is a critical marker for immature neurons during the early stages of neuronal differentiation, used to evaluate the integrity of neurogenesis [65]. Therefore, its reduced expression in this context clearly suggests that maternal obesity may disrupt fetal hippocampal neurogenesis and neuronal integrity [64].

## 4 Functional connectivity data in humans

In humans, there is only limited research investigating the effects of prenatal maternal obesity on infant neurodevelopment [66]. Evidence suggests that maternal metabolic health influences early brain organization, possibly through functional architecture and neurodevelopmental trajectories of the newborn brain [66,67,[67], [67], [68], [69], [70], [71]]. Several studies have linked maternal body mass index (BMI) with reduced cortical thickness and altered functional connectivity in neonatal frontal brain regions [70,72,73]. Using resting-state functional magnetic resonance imaging data, in 2-week-old infants whose mothers had high BMI, Salzwedel AP et al. detected functional connectivity alterations in four domains critically implicated in adult obesity pathophysiology, including sensory cue processing (especially visual cues), reward processing, cognitive control, and motor control [69]. Li et al. reported that, in 2-week-old infants, functional connectivity strength in dorsal anterior cingulate cortex regions to the prefrontal network was negatively correlated to maternal fat mass percentage measured at early pregnancy [68]. Using diffusion tensor imaging (DTI), Rosberg et al. documented that pre-pregnancy maternal body weight influences reward-related striatal brain areas of the offspring during *in utero* development, with higher maternal pre-pregnancy BMI being associated with higher mean diffusivity in the infant's left caudate nucleus [66]. To evaluate brain white matter development in 2-week-old healthy newborns from uncomplicated singleton pregnancies, Ou X et al. used DTI to compare fractional anisotropy (FA), a sensitive measure of white matter integrity with higher FA values representing better development, between either normal-weight or otherwise healthy mothers with BMI ≥30 kg/m^2^, at conception. According to this study, infants from maternally obese mothers had lower FA values in whole brain white matter as well as in all major tracts including the inferior fronto-occipital longitudinal fasciculus, the superior longitudinal fasciculus, the external and anterior internal capsule, the forceps minor, the genu of the corpus callosum, the fornix, and the anterior and superior corona radiata [67]. Furthermore, a DTI analysis in preterm neonates born to high-BMI mothers showed that maternal BMI was associated with a lower white matter volume and altered microstructure of the uncinate fasciculus [74]. Collectively, these findings suggest that some pre-conception or *in utero* factor(s) in high-BMI mothers disrupt white matter developmental trajectories in infants. Recently, increased maternal pre-pregnancy BMI has been reported to negatively correlate with the stability of a functional cortical network encompassing the superior frontal, superior parietal, and temporal regions in the offspring [75]. Ultrasound images from 166 pregnancies were used for cerebellar growth rates measurements. Pre-pregnancy and first trimester BMI were significantly associated with decreased cerebellar growth trajectories [76].

## 5 Mechanistic insights

During development, the ontogenetic expansion of neural processes and the arborization of developing neurons are orchestrated by a sophisticated array of cellular and molecular mechanisms which influence growth rate, direction, and branching [77]. The mechanisms through which maternal obesity is associated with neurodevelopmental alterations are unclear. It has been suggested that maternal cytokine levels, oxidative stress, and inflammation in the placenta may interact with fetal neurodevelopment, possibly through changes to glia cytoarchitecture and epigenetic modifications [78].

Maternal cytokines, such as interleukin 6 (IL-6), tumor necrosis factor alpha (TNF-alpha), and transforming growth factor beta (TGF-beta), as well as adipokines, such as leptin and adiponectin, have direct impact on placental function by modulating nutrient delivery to the fetus [79]. Cytokines, such as IL-6, TNF-alpha, and TGF-beta are involved in various aspects of neural development from initial central nervous system (CNS) formation to synaptogenesis [78,80]. Beyond its inflammatory role, IL-6 regulates the self-renewal of neuroepithelial cells and axon development [80]. IL-6 also negatively modulates neurite extension in cultured hypothalamic tissue. Notably, offspring of obese rodent models show reduced neurite growth in the arcuate nucleus and impaired development of NPY arcuate-paraventricular projections [81]. These data underscore the potential for obesity-induced cytokine surges to disrupt the formation of hypothalamic circuits. Furthermore, TNF-alpha is another cytokine involved in neurite outgrowth [77]. However, in the context of developmental programming, elevated TNF-alpha expression resulting from maternal obesity has been suggested to reduce axon growth within the sympathetic nervous system [78].

This inflammatory disruption extends to other critical neurodevelopmental pathways. TGF-beta is essential for CNS development from the earliest stages of embryogenesis through adulthood [82]. Co-expressed with sonic hedgehog in early embryonic structures such as the notochord and floor plate, TGF-beta is critical for the induction, survival, and differentiation of midbrain dopaminergic neurons, while also regulating their axonal growth and synaptic electrophysiology [[83], [84], [85]]. Moreover, intact TGF-beta signaling is indispensable for the structural and functional maturation of astrocytes and microglia [78,86]. Given its foundational role in establishing neural and glial architecture, the deregulation of TGF-beta provides a crucial mechanistic link between maternal metabolic stress and altered fetal brain development. Indeed, recent evidence demonstrates that offspring from mothers exposed to a high-fat diet exhibit aberrantly elevated hypothalamic TGF-beta expression, impaired LKB1-TGF-beta1 signaling, and deregulated energy homeostasis networks [87].

A recent study examined how maternal high-fat diet affects embryonic cerebral cortex development in mice, with specific focus on sex-dependent placental cytokine changes. Carrazana et al. suggest that TNF-alpha elevation in female placentas from high-fat diet-exposed mothers may impair neural progenitor proliferation, while IL-6 reduction in males could compromise neurogenesis, as IL-6 has neurotrophic roles and stimulates embryonic forebrain precursor cell expansion [88]. Consequently, disruption of these critical morphogenic and inflammatory signals by maternal obesity may profoundly impair neurodevelopmental trajectories in a sex-specific manner.

It is well established that central regulation of energy balance is mediated by complex and anatomically distributed neuronal circuits. However, the role of the non-neuronal cellular compartment is still unclear. Being fundamental for brain development non-neuronal cells, including astrocytes, microglia, oligodendrocytes, and ependymal cells/tanycytes, mediate several aspects of neuronal proliferation, survival, metabolism, and function [89,90]. Cytokine-driven changes in glia represent another mechanism for disrupted neurodevelopment [78]. In rodents, maternal obesity can induce a pro-inflammatory phenotype in microglia postnatally, with increased levels of microglial activation marker CD11b within the hippocampus [91]. It has been suggested that metabolic disruptions, such as hyperleptinemia, and lipid droplet–containing microglia with impaired phagocytic function and pro-inflammatory secretome might impair neurodevelopment in the offspring [78,92].

The effects of maternal overnutrition on fetal brain development are also mediated by dysregulated epigenetic mechanisms [93]. Maternal metabolic disturbances, characterized by elevated cytokines, oxidative stress, and inflammation, interact with fetal neurodevelopment through two converging pathways: DNA methylation and histone modification [78,93]. These modifications serve as a molecular memory of the intrauterine environment, potentially altering gene expression programs critical for brain architecture and function.

In rodent models, maternal high-fat diet during pregnancy and lactation has been shown to induce global DNA hypomethylation in the VTA, nucleus accumbens, hypothalamus, and prefrontal cortex, as well as specific hypomethylation of promoter regions for the *DAT*, *MOR,* and *PENK* promoters in the hypothalamus and nucleus accumbens, and significant hypomethylation of *MOR* in the prefrontal cortex [55]. This hypomethylation correlates with upregulated expression of these genes in male offspring, driving an increased preference for palatable foods and altered hedonic feeding behaviors [55].

Interestingly, human studies utilizing genome-wide DNA methylation analyses of umbilical cord tissue, a surrogate for fetal brain development, have revealed epigenetic patterns linked to maternal adiposity. Specifically, maternal fat mass in pregnancy correlates with the hypermethylation of genes essential for neurogenesis and white matter maturation, including *NKX2-1, LMX1A, FOXA2*, and *HES1* [67]. Conversely, other key neuronal genes, such as *GSX2, IGF-1R,* and *FYN*, exhibited hypomethylation associated with higher maternal adiposity [67]. This hypermethylation of pathways governing axonogenesis and oligodendrocyte differentiation is associated with reduced white matter integrity in human neonates [67]. These translational findings indicate that maternal obesity prompts a complex gene-specific epigenetic landscape in humans, spanning both repressive (hypermethylation) and activating (hypomethylation) changes, which differs from the more uniform hypomethylation observed in reward-related pathways in rodent models.

Beyond DNA methylation, maternal diet significantly impacts histone modifications, which regulate chromatin accessibility and gene transcription. It has been suggested that the expression of sirtuin 1 (SIRT1), a class III histone deacetylase involved in fat metabolism and glucose homeostasis regulation, is decreased in rodent fetal organs in response to maternal high-fat diet [94,95]. Furthermore, SIRT1 knockdown via siRNA attenuated neuronal differentiation and disrupted the migration of neural progenitor cells into the embryonic cortical plate [93,96]. Reduced SIRT1 activity leads to hyperacetylation of histones (e.g., H3 and H4), altering the transcription of genes involved in neuronal survival and being associated with defects in synaptic plasticity [97].

In addition to SIRT1-mediated deacetylation, maternal obesity disrupts histone methylation patterns. Recent evidence from a maternal high-fat diet rat model reveals that metabolic stress activates AMP-activated protein kinase, which phosphorylates the histone methyltransferase EZH2 at Thr311, thereby suppressing its catalytic activity [98]. This phosphorylation leads to a significant reduction in the repressive histone mark H3K27me3 at promoters of key developmental genes in the embryonic cortex. Consequently, EZH2 target genes become transcriptionally derepressed, including those that should remain silent during neurogenesis, which shifts cell fate toward astrogliogenesis and impairs proper cortical differentiation [98]. These mechanisms underscore how maternal metabolic signals can directly reprogram chromatin architecture, creating a permissive environment for aberrant gene expression during critical periods of neurogenesis.

## 6 Discussion

**Figure 1:**
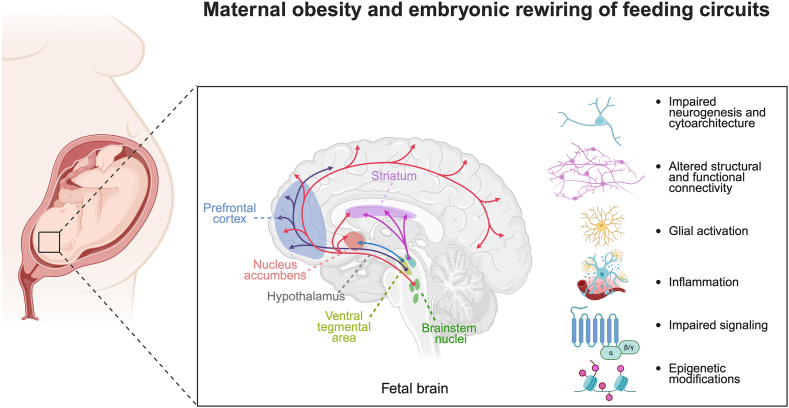
Embryonic rewiring of distributed feeding circuits in response to maternal obesity. An obesogenic maternal environment disrupts not only the hypothalamus of the developing fetus but also extra-hypothalamic regions, including brainstem interoceptive nuclei and mesocorticolimbic reward-control systems, which undergo significant cytoarchitectural and functional reprogramming, mediated by neuroinflammation, impaired orexigenic and anorexigenic signaling, and epigenetic alterations, ultimately compounding the offspring long-term risk for metabolic dysfunction and obesity. Created in https://BioRender.com.

The developing brain is vulnerable to environmental conditions during pregnancy. Maternal overnutrition influences intrauterine metabolic programming in the offspring and may affect trajectories of neurologic development, ultimately compromising the structural and functional characteristics of hypothalamic, brainstem, and mesocorticolimbic circuits. The impact of maternal obesity on fetal neurodevelopment may be mediated by inflammatory, placental, and epigenetic mechanisms, leading to dysregulation of offspring metabolic health (Fig. 1) [99]. Although hypothalamic reprogramming has dominated research, emerging evidence supports a broader, distributed model involving both brainstem interoceptive circuits and mesocorticolimbic reward-control systems. Experimental findings demonstrate that maternal high-fat diet exposure can alter both cytoarchitecture and function of neurons and non-neuronal compartments within the brainstem and reward circuitry. Data in humans are scarce and are primarily based on neuroimaging findings linking maternal adiposity to altered neonatal brain network connectivity and cortical architecture. However, these findings do not establish direct causality, and the association between maternal adiposity and neurodevelopmental alterations may be subject to confounding variables, such as socioeconomic factors, maternal stress, and genetic inheritance [[66], [67], [68], [69], [70], [71],^73^,^75^].

It is important to distinguish between maternal obesity (the metabolic state) and maternal high-fat diet (the nutritional insult), as their programming outcomes may differ. For instance, embryo transfer models in mice have demonstrated that pre-pregnancy obesity independent of gestational obesity is sufficient to induce persistent changes in the reward circuitry of the offspring [57]. Regardless of the specific trigger, these neurodevelopmental alterations are categorized as maternal effects, as they are established *in utero* and during lactation, manifesting before the offspring transition to independent feeding [[55], [56], [57],^91^,^99^].

It is crucial to acknowledge that the effects of maternal high-fat diet are not static but evolve over time. Evidence highlights critical time-dependent differences in these neurochemical signatures. For instance, expression profiles at E17.5 often deviate from adult outcomes; MOR and PENK transiently decrease in the male embryonic forebrain at E17.5, even though they are increased in adulthood [57]. Additionally, in male, but not female, rats, while MOR expression correlates positively with fat intake during the immediate post-weaning period (6 weeks), this relationship is reversed by 3 months of age [54,61]. These distinct ontogenetic shifts suggest that the window of vulnerability is highly specific and distinct from the acute responses observed in adults. However, the permanence of this developmental programming remains contested [2]. Future long-term studies are essential to clarify whether these ontogenetic shifts represent permanent neurological adaptations or transient responses that may reverse later in adulthood.

Although most studies investigating the influence of maternal obesity on neuronal circuits have focused on male rodent offspring, emerging evidence highlights a sexual dimorphism in the effects of maternal obesity on offspring. In mouse models from mothers exposed to high-fat diets, sexually dimorphic placental cytokine profiles may contribute to observed neurodevelopmental differences [88]. Grissom et al. [57] demonstrated that offspring reward circuitry programming exhibits profound sexual dimorphism: while adult males exposed to pre-pregnancy or gestational obesity showed elevated MOR and PENK expression across the mesocorticolimbic system, females generally displayed a reduced expression profile. Similarly, DAT levels exhibited a sex-specific divergence, with significant increases in adult males but decreases in females, suggesting unique sensitivity to the maternal metabolic milieu. This dimorphism extends to the postnatal period. Exposure to maternal high-fat diet during lactation constitutes a potent maternal effect that increases early-life adiposity and intensifies long-term metabolic risk, predominantly in males, whereas females appear largely protected from these long-term consequences [[99], [100], [101]]. Consistently, maternal high-fat diet during lactation prompts a fundamental restructuring of basal ganglia circuitry, establishing sex-specific phenotypes that persist into adulthood. Remarkably, these neurochemical shifts translate into sexually dimorphic behavioral outcomes, manifesting as hyperlocomotion in males and increased reward-seeking consumption in females [16]. Parallel to reward signaling, sex-specific programming is also evident in autonomic regulation within the brainstem. Specifically, maternal high-fat diet triggers a male-specificdown-regulation of medullary mGlu_2/3_R and GABA_A_R expression, suggesting that increased sympathetic tone and autonomic imbalance are sex-dependent consequences of maternal programming [49].

## 7 Future directions

Despite the emerging evidence linking maternal overnutrition and obesity to the rewiring of extra-hypothalamic circuits, several fundamental questions remain unanswered. Does the embryonic rewiring of reward and brainstem circuits persist into late adulthood, or can it be mitigated by early life nutritional interventions? Beyond neuronal changes, what is the precise contribution of non-neuronal cells, such as astrocytes and microglia, in the programming of extra-hypothalamic regions? The mechanisms by which signals about adverse maternal metabolic status are conveyed to brainstem and mesolimbic dopamine reward circuits that regulate motivated feeding behavior remain largely unknown. Are intrauterine programming and altered neurodevelopmental processes associated with maternal inflammation induced by obesity or with the direct effects of maternal diet? Furthermore, how do postnatal factors, such as maternal stress or breastfeeding —breast milk of high-BMI mothers has pro-inflammatory properties and decreased levels of fatty acids and carotenoids, which are critical to neurodevelopment [102]— further modulate these neurodevelopmental trajectories?

Future studies incorporating a wider range of covariates, maternal and placental transcriptomic analyses, and long-term anthropometric and behavioral follow-up in offspring would strengthen causal inference. Further research is necessary to elucidate the mechanisms through which exposure to maternal obesity influences brainstem energy balance-related nuclei, such as the parabrachial nuclei and the area postrema, as well as the interactions between hypothalamic, brainstem, and mesocorticolimbic neuronal networks in the offspring. Additionally, because the investigation of the brainstem using structural or functional magnetic resonance imaging is technically challenging, especially in neonates, the broader adoption of ultrahigh-field MRI scanning may permit more precise investigation of brainstem nuclei connectivity, elucidating the neuroanatomy and neurophysiology of feeding-related neural circuits [103]. Finally, the expanding implementation of new techniques, such as spatial and single-cell transcriptomics in rodent models, may shed new light on the complex signaling networks that are affected by *in utero* nutritional stresses.

## CRediT authorship contribution statement

**Dionysios V. Chartoumpekis:** Writing – review & editing, Resources, Methodology, Investigation, Formal analysis, Data curation, Conceptualization. **Aristea Psilopanagioti:** Writing – review & editing, Writing – original draft, Visualization, Software, Project administration, Methodology, Investigation, Formal analysis, Data curation, Conceptualization.

## Informed consent statement

Not applicable.

## Consent for publication

Not applicable. This study involves only computational analyses of publicly available data and does not include any studies with human participants or animals.

## Institutional review board statement

Not applicable.

## Generative AI statement

The authors declared that generative AI was not used in the creation of this manuscript.

## Funding

None.

## Declaration of competing interest

The authors declare that they have no competing financial interests or personal relationships that could have appeared to influence the work reported in this paper.

## Data Availability

No data was used for the research described in the article.
